# MRI-based assessment of fetal brain gyrification: Reliability across two methods

**DOI:** 10.1016/j.ynirp.2026.100395

**Published:** 2026-08-12

**Authors:** Lotte Meijerink, Yvette Stam, Fieke Terstappen, Thomas Alderliesten, Manon Benders, Wouter P. Nieuwenhuis, Rutger A.J. Nievelstein, Mireille Bekker

**Affiliations:** a Department of Obstetrics, Division Woman & Baby, University Medical Center Utrecht, Utrecht University, Utrecht, the Netherlands; b Department of Obstetrics, St. Elisabeth Hospital, Tilburg, the Netherlands; c Department of Neonatology, Division Woman & Baby, University Medical Center Utrecht, Utrecht University, Utrecht, the Netherlands; d Department of Radiology & Nuclear Medicine, Division Imaging & Oncology, University Medical Center Utrecht, Utrecht University, Utrecht, the Netherlands

**Keywords:** Fetal gyrification, Fetal MRI, Maturation assessment

## Abstract

**Objective:**

To explore the reliability of atlas-based assessment of fetal gyrification on MRI, given the absence of a standardized method to assess cerebral gyrification on fetal imaging and the potential of recently published gestational-age specific fetal brain atlases including high-resolution anatomical detail.

**Materials and methods:**

Fetal MRI scans (n = 20; gestational age 26 + 0 to 33 + 1 weeks) were retrospectively randomly selected, including 10 healthy controls and 10 cases with early-onset fetal growth restriction, of which 5 were suspected of delayed fetal gyrification based on comparison with an atlas from 2004. Two experienced pediatric neuroradiologists, independently and blinded to clinical condition, assessed each scan using two approaches: (1) a 2D template from the 3D Gholipour atlas, and including measurements of Sylvian fissure angle and Pistorius scoring, and (2) a full 3D atlas-based evaluation.

**Results:**

Intraclass correlation coefficients (ICCs) between radiologists' estimates of GA and the actual GA were above 0.940 for both methods. Inter-rater ICCs were 0.937 for method 1 and 0.932 for method 2. In contrast, ICCs for the Sylvian fissure angle or the Pistorius score for the left and right side were low (0.393, 0.649, 0.098 and 0.131, respectively). Determining delayed gyrification proved challenging, with Cohen's Kappa values between 0.22 and 0.64 between methods and radiologists.

**Conclusion:**

Gestational age based on gyrification can be estimated with high reliability, both in agreement with actual GA and between raters, using both 2D and 3D MRI templates from the Gholipour fetal brain atlas. These findings support the reliability of standardized fetal brain templates for assessment of fetal gyrification within this single-center cohort. Reproducibility across centers, scanners and clinical settings remains to be established.

## Highlights

• Supports standardized atlas use for fetal brain maturation assessment.
• Gestational age can be accurately estimated from 2D and 3D MRI atlases.
• Conventional Sylvian fissure and Pistorius scores show poor consistency.
• Detecting delayed gyrification on fetal MRI remains challenging.

## 1 Introduction

Gyrification, the process of cortical folding, is an important part of fetal brain development. Abnormal gyrification has been linked to various disorders and syndromes and has also been observed in cases of fetal growth restriction (FGR), highlighting the importance of accurate prenatal assessment (Wyburd et al., 2021; Namburete et al., 2023; Dubois et al., 2008).

Prenatal gyrification can be assessed with ultrasound or MRI, both with their own strengths and limitations. Ultrasound is widely used in clinical practice for assessing fetal brain development due to its accessibility and real-time imaging capabilities. However, ultrasound has some limitations in evaluating the fetal cortex like reduced image quality caused by fetal position, maternal habitus, or shadowing effects from the fetal skull (Gholipour et al., 2014). Moreover, cortical evaluation is subject to interobserver variation and requires sufficient expertise in fetal neurosonography (Ginsberg et al., 2021). One of the most common methods for assessing cerebral maturation on ultrasound is examining the sylvian fissure by measuring the angle and applying the Pistorius score which divides cortical folding into five categories by gestational age (GA) (Pistorius et al., 2010; Poon et al., 2019; Pooh et al., 2019).

Fetal magnetic resonance imaging (MRI) provides higher contrast resolution with superior tissue differentiation and is less influenced by fetal position and maternal characteristics. As a result, MRI is often used when there are concerns about fetal cortical development on ultrasound (Bekker et al.). Early-onset fetal growth restriction (FGR) has been associated with delayed gyrification (Meijerink et al., 2026). This association motivated the present study, which evaluates the reliability of atlas-based gyrification assessment methods that could be used to systematically investigate this association in future work.

Given the recent advances in GA-specific fetal brain atlases providing high-quality, anatomically detailed reference images, there is now an opportunity to develop more reproducible assessment techniques (Gholipour et al., 2017; Ciceri et al., 2024a). Several quantitative approaches have nonetheless demonstrated accurate gestational age prediction from cortical morphology, including curvature- and shape-based descriptors of cortical folding, even in pathological populations (Ciceri et al., 2024b; Wu et al., 2015; Hong et al., 2021; Hu et al., 2013). While these methods offer objective, reproducible metrics of gyrification, they typically require 3D surface reconstruction and specialized post-processing pipelines that are not yet readily available in routine clinical workflows (Bekker et al.; Ciceri et al., 2024b; Wu et al., 2015; Hong et al., 2021; Hu et al., 2013). In contrast, the present study evaluates a pragmatic, visual, atlas-based approach that radiologists can apply directly within existing clinical PACS environments, positioning it as a complementary, clinically accessible alternative to these quantitative techniques rather than a replacement for them (Bekker et al.; Ciceri et al., 2024b; Wu et al., 2015; Hong et al., 2021; Hu et al., 2013).

The aim of the study is to compare two atlas-based methods for assessing fetal gyrification on MRI and to evaluate their reliability as a step towards establishing a standardized and reproducible prenatal cortical evaluation. We hypothesize that atlas-based assessment of fetal cortical gyrification on MRI will demonstrate high reliability and agreement with actual GA, across methods and between raters. However, despite high reliability, classifying delayed gyrification will prove challenging.

## 2 Methods

### 2.1 Selection of participants

Twenty fetal MRI scans were retrospectively selected, including 10 healthy controls and 10 early-onset FGR cases, 5 of which were suspected of delayed gyrification by the pediatric radiologist (RJN) based on comparison with an atlas from 2004 in clinical setting (Garel, 2004). The FGR cases were part of a prospective monocenter observational study at the University Medical Center Utrecht (June 2022–June 2024). Inclusion criteria specified early-onset FGR with brain-sparing. Ethical approval was obtained prior to study initiation (protocol: 22–552; April 7, 2022). The healthy controls were selected from the YOUth longitudinal cohort after the data request was approved (Onland-Moret et al., 2020; Meijerink et al., 2023).

FGR was defined according to the Delphi consensus definition as an estimated fetal weight and/or abdominal circumference below the tenth percentile in combination with evidence of brain-sparing. Brain-sparing was diagnosed using Doppler ultrasound, defined as an umbilical artery pulsatility index (PI) above the 95th percentile together with a middle cerebral artery PI below the 5th percentile or a cerebroplacental ratio below 1 (Gordijn et al., 2016). The FGR scans were acquired between 24 and 32 weeks of gestation while control scans were obtained at approximately 30 weeks’ gestation as part of the YOUth study (Meijerink et al., 2023).All data were pseudonymized, ensuring that clinical status (FGR vs. control) remained blinded throughout the analysis.

### 2.2 MR imaging protocol

All MRI scans were performed on a 3.0 T MRI (Philips Intera, Philips Healthcare, Best, The Netherlands). Pregnant women were scanned without sedation in left lateral tilt position using a transmit coil. The standardized protocol included T2-weighted single-shot turbo spin echo sequences acquired in three orthogonal planes (axial, sagittal, and coronal), with an in-plane resolution of 1.25 × 1.25 mm, a slice thickness of 2.5 mm with an overlapping slice gap of 1.25 mm, an echo time of 180 ms, and a repetition time of 5321 ms. No 3D or super-resolution reconstruction was performed; all assessments were based on the multi-planar 2D acquired slices.

### 2.3 Methods for maturation assessment

**Fig. 1:**
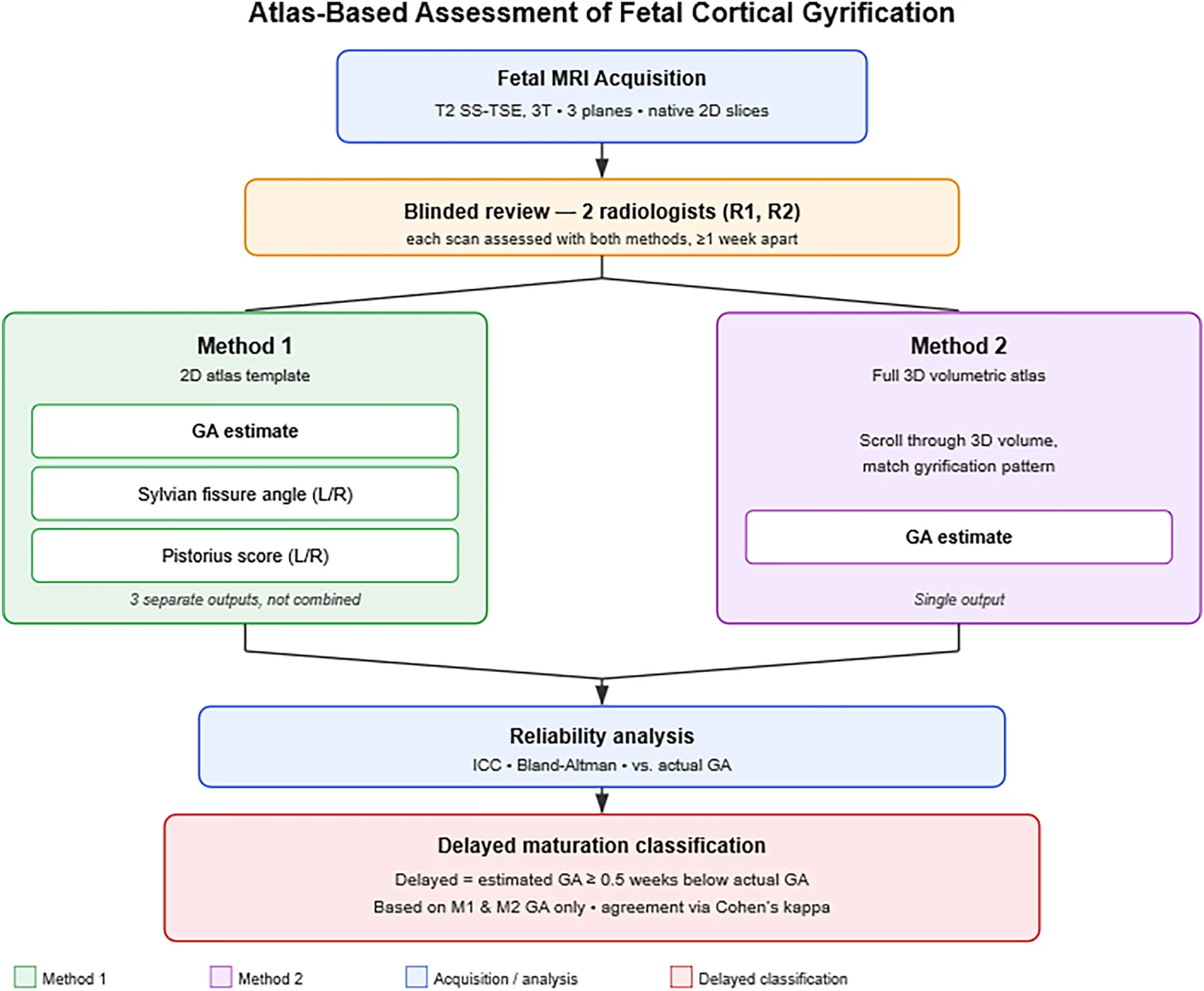
Schematic workflow of atlas-based assessment of fetal cortical gyrification on MRI GA = gestational age; M1 = Method 1 (2D template-based assessment); M2 = Method 2 (3D volumetric atlas-based assessment); R1 = Radiologist 1; R2 = Radiologist 2; L = left; R = right; ICC = intraclass correlation coefficient.

Two pediatric radiologists (RJN and WN; 25 and 5 years of experience), independently evaluated the fetal MRI scans blinded for all clinical information except for gestational age to mimic clinical practice. To reduce measurement and confirmation bias, both raters were blinded to each other's assessments and to the results of the alternate method throughout the study. The initial assessment of brain maturation (method 1, M1) included three separate outputs: estimation of gestational age (weeks + days) using the 2D template from the 3D fetal brain atlas developed by Gholipour (Gholipour et al., 2017), and measuring the Sylvian fissure angle in the coronal plane and applying the Pistorius score, each assessed separately for the left and right hemispheres (Pistorius et al., 2010; Pooh et al., 2019) (Fig. 1). For the template-based GA estimation, radiologists visually compared the subject's MRI across all three orthogonal planes (axial, coronal, and sagittal) against the corresponding 2D template images from the atlas and subjectively selected the whole-week template judged to be the best visual match. When a case appeared intermediate between two adjacent whole-week templates, radiologists were permitted to interpolate and record a half-week estimate. No structured scoring criteria or landmark checklist were used. The Pistorius score, originally developed for ultrasound, classifies cortical folding into five categorical stages by gestational age range. It was applied here in its original categorical form and was not converted into a continuous GA estimate. For MRI-based assessment, the transversal plane of the T2-weighted images was used for the Pistorius scoring, while the coronal plane was used for Sylvian fissure angle measurement. The three M1 outputs (template-based GA, Sylvian fissure angle, and Pistorius score) were recorded independently and were not combined into a single composite GA estimate. Only the template-based GA estimate was used as the primary outcome for the reliability analyses, while the Sylvian fissure angle and Pistorius score were evaluated as secondary, exploratory markers. After an interval of at least one week, the same scans were re-evaluated at random using the second method. The second method implemented the full use of the 3D atlas by Gholipour to estimate gestational age (weeks + days) based on gyrification by scrolling through the volumetric data (Gholipour et al., 2017). Radiologists visually scrolled through the whole-brain 3D atlas volume and subjectively selected the whole-week atlas volume judged to provide the best overall gyrification match to the subject's scan, again interpolating to a half-week estimate for intermediate cases. As with M1, no quantitative registration metric or structured scoring criterion was used. The order in which method 1 and method 2 were performed was not randomized. This fixed sequence is acknowledged as a potential source of order bias and is addressed further in the Limitations section. In total, each fetal MRI scan was independently assessed four times (twice by each radiologist, once using each method), with no additional repeated runs. To mitigate the influence of rater experience asymmetry (25 vs. 5 years) on reliability estimates, results are reported separately for each radiologist's agreement with actual GA reliability, across methods and between raters, allowing assessment of whether findings were consistent across experience levels. Delayed maturation was dichotomized and defined as an estimated GA≥0.5 weeks younger than the actual GA during the MRI, based solely on the atlas-derived GA estimates from method 1 (2D template) and method 2 (3D volumetric atlas); the Sylvian fissure angle and Pistorius score were not used in the delayed-maturation classification. This cut-off was chosen because the reference atlas presents gestational age in whole-week intervals; a discrepancy of ≥0.5 weeks therefore reflects at least a half-template shift relative to the reference standard (see Fig. 1).

### 2.4 Data management and pre-processing

All fetal MRI scans were anonymized prior to review and assigned a unique study identifier; no protected health information was accessible to either rater during assessment. GA estimates, Sylvian fissure angle measurements, and Pistorius scores were recorded directly into a structured Castor form (Castor EDC, Amsterdam, The Netherlands). Images were reviewed using PACS. No image pre-processing was applied.

### 2.5 Statistical analysis

Agreement between the actual GA during the MRI to the GA estimated by Radiologist 1 (R1) using both method 1 (M1) and method 2 (M2), and subsequently by Radiologist 2 (R2) using the same two methods, was assessed with intraclass correlation coefficients (ICCs). In addition, the two methods were compared within each radiologist, and the same method was compared between the two radiologists. ICCs were calculated using a two-way random effects model with absolute agreement, based on single measurement. Statistical analyses were performed in SPSS version 32.0.0.0 (IBM Corp., Armonk, NY). Bland-Altman plots were generated for all comparisons to further evaluate agreement and to identify any systematic bias between methods or raters.

The reliability for assessing delayed maturation was then assessed for M1 and M2 by R1, subsequently for both methods by R2, and then between the radiologists with a Cohen's kappa (McHugh, 2012). Additionally, a sensitivity analysis was performed using a wider 1-week threshold rather than 0.5 weeks for delayed maturation. As this study focused on estimation of agreement and reliability rather than formal hypothesis testing across multiple independent comparisons, no correction for multiple comparisons was applied.

## 3 Results

### 3.1 Patient characteristics

Gestational age at the time of scanning was significantly lower in the FGR group compared to healthy controls (median [IQR] = 29.1 [3.0] vs 31.7 [2.9] weeks, *p* < 0.01), which is expected since fetal MRI in FGR is typically performed in a clinical setting shortly after the diagnosis of brain-sparing, whereas healthy controls are scanned in research setting around 30 weeks’ gestation. Maternal age, BMI, and parity did not differ significantly between groups (*p* = 0.90, 0.55, and 0.52, respectively). However, the proportion of female fetuses was lower in the FGR group (1/10 vs 6/10, *p* = 0.02). When comparing fetuses with delayed versus non-delayed maturation in the FGR group, no significant differences were found in gestational age at scan, maternal age, BMI, parity, or fetal sex (all p > 0.29).

### 3.2 Agreement with GA, and inter-rater and inter-method variability

R1 agreement between the estimated and actual GA was excellent, with an intraclass correlation coefficient (ICC) of 0.975 for M1 (2D template) and 0.982 for M2 (3D atlas). Similarly, for R2, the ICCs were 0.948 for M1 and 0.940 for M2. When comparing the two methods within each radiologist, strong agreement was observed. The ICC between M1 and M2 for R1 was 0.946, and for R2 it was 0.968, suggesting that both approaches yielded comparable estimates of GA within raters. Interrater agreement was also high. For M1, the ICC between R1 and R2 was 0.937, while for M2 the ICC was 0.932.

**Fig. 2:**
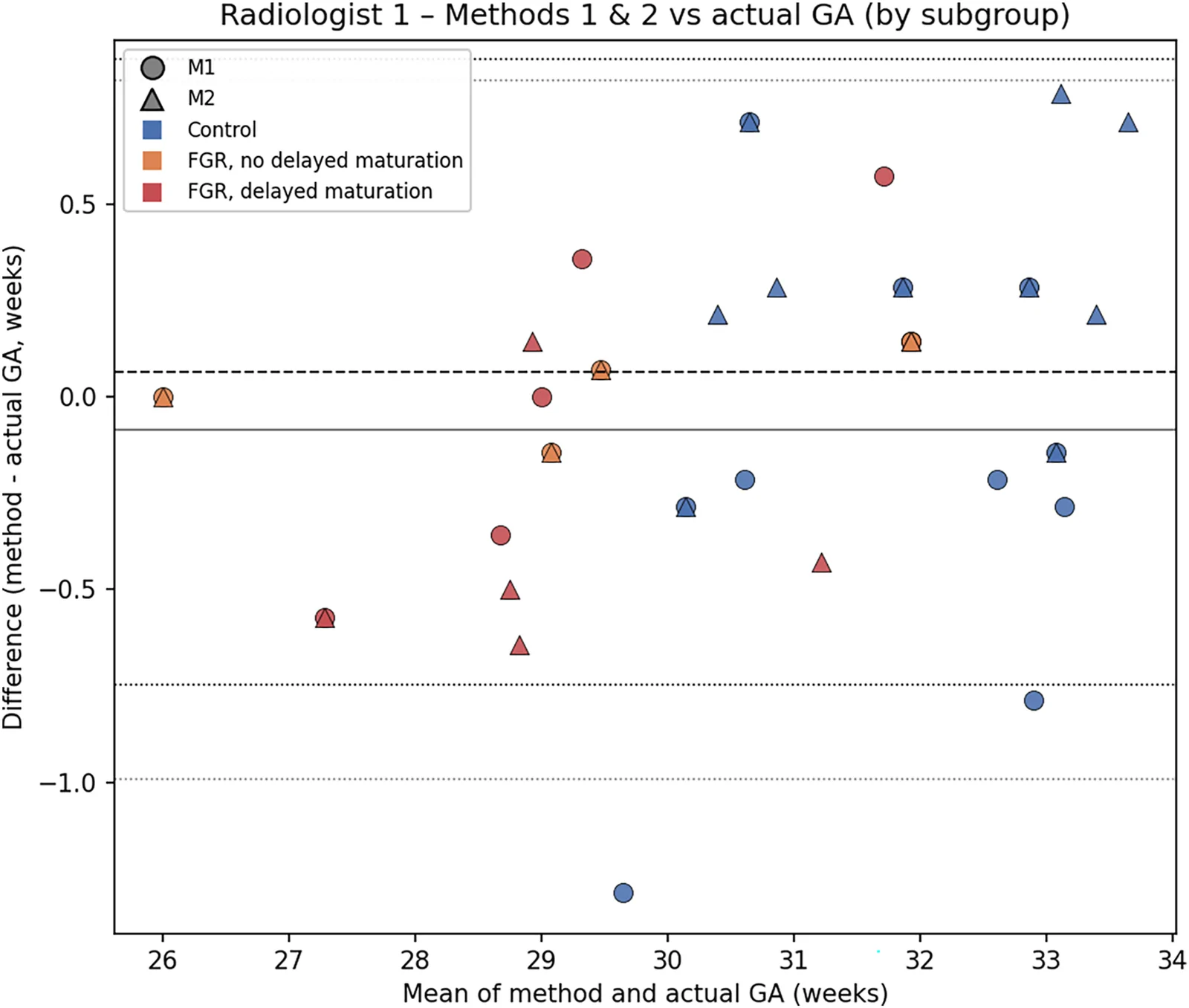
Radiologist 1 – Method 1 and 2 versus actual gestational age at MRI GA = gestational age; Method 1 = estimated gestational age in weeks based on the 2D template from the 3D fetal brain atlas developed by Gholipour (Gholipour et al., 2017); Method 2 = estimated gestational age in weeks based on the full 3D fetal brain atlas developed by Gholipour (Gholipour et al., 2017); The solid lines indicate mean bias; dashed lines indicate limits of agreement (±1.96 standard deviation).

**Fig. 3:**
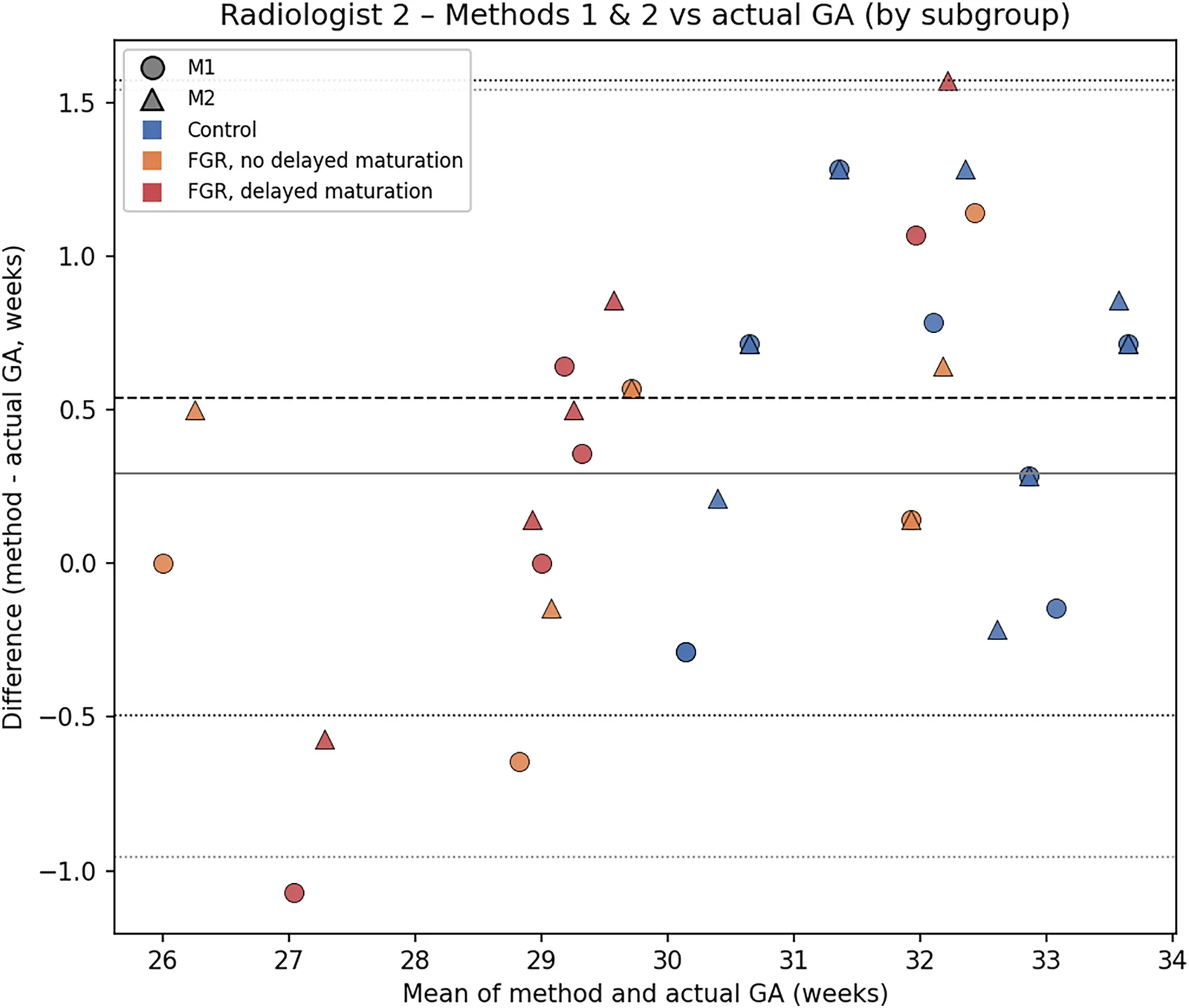
Radiologist 2 – Method 1 and 2 versus actual gestational age at MRI GA = gestational age; Method 1 = estimated gestational age in weeks based on the 2D template from the 3D fetal brain atlas developed by Gholipour (Gholipour et al., 2017); Method 2 = estimated gestational age in weeks based on the full 3D fetal brain atlas developed by Gholipour (Gholipour et al., 2017); The solid lines indicate mean bias; dashed lines indicate limits of agreement (±1.96 standard deviation).). A proportional bias was observed for R2, with larger positive differences (overestimation) at higher mean GA; this pattern was significant within the FGR subgroup (M1: r = 0.69, p = 0.03; M2: r = 0.51, p = 0.13) but not among controls (M1: r = 0.25, p = 0.55; M2: r = 0.01, p = 0.99), suggesting it was driven predominantly by the FGR cases.

**Fig. 4:**
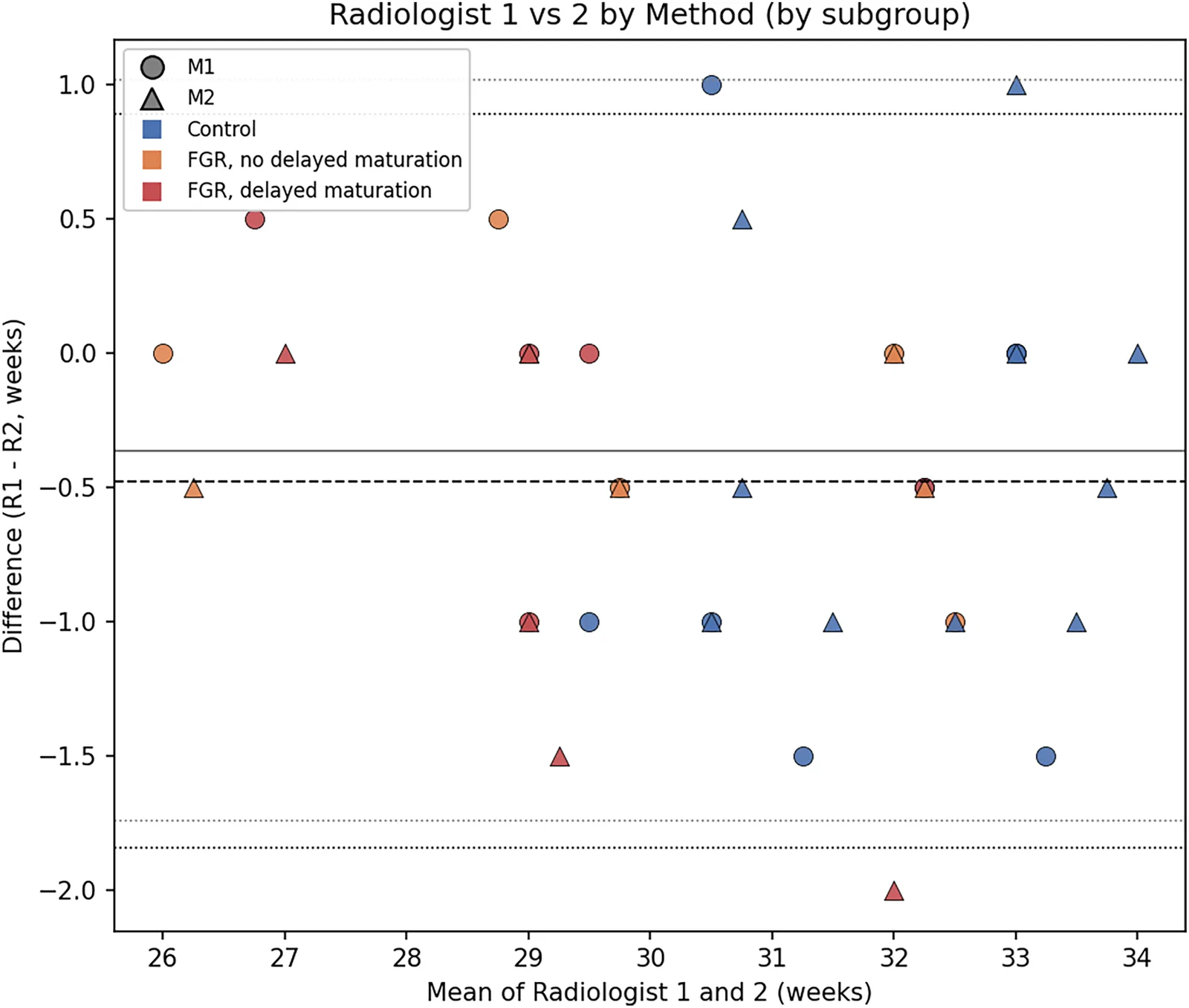
Radiologist 1 vs Radiologist 2 by Method Method 1 = estimated gestational age in weeks based on the 2D template from the 3D fetal brain atlas developed by Gholipour (Gholipour et al., 2017); Method 2 = estimated gestational age in weeks based on the full 3D fetal brain atlas developed by Gholipour (Gholipour et al., 2017); The solid lines indicate mean bias; dashed lines indicate limits of agreement (±1.96 standard deviation).

**Fig. 5:**
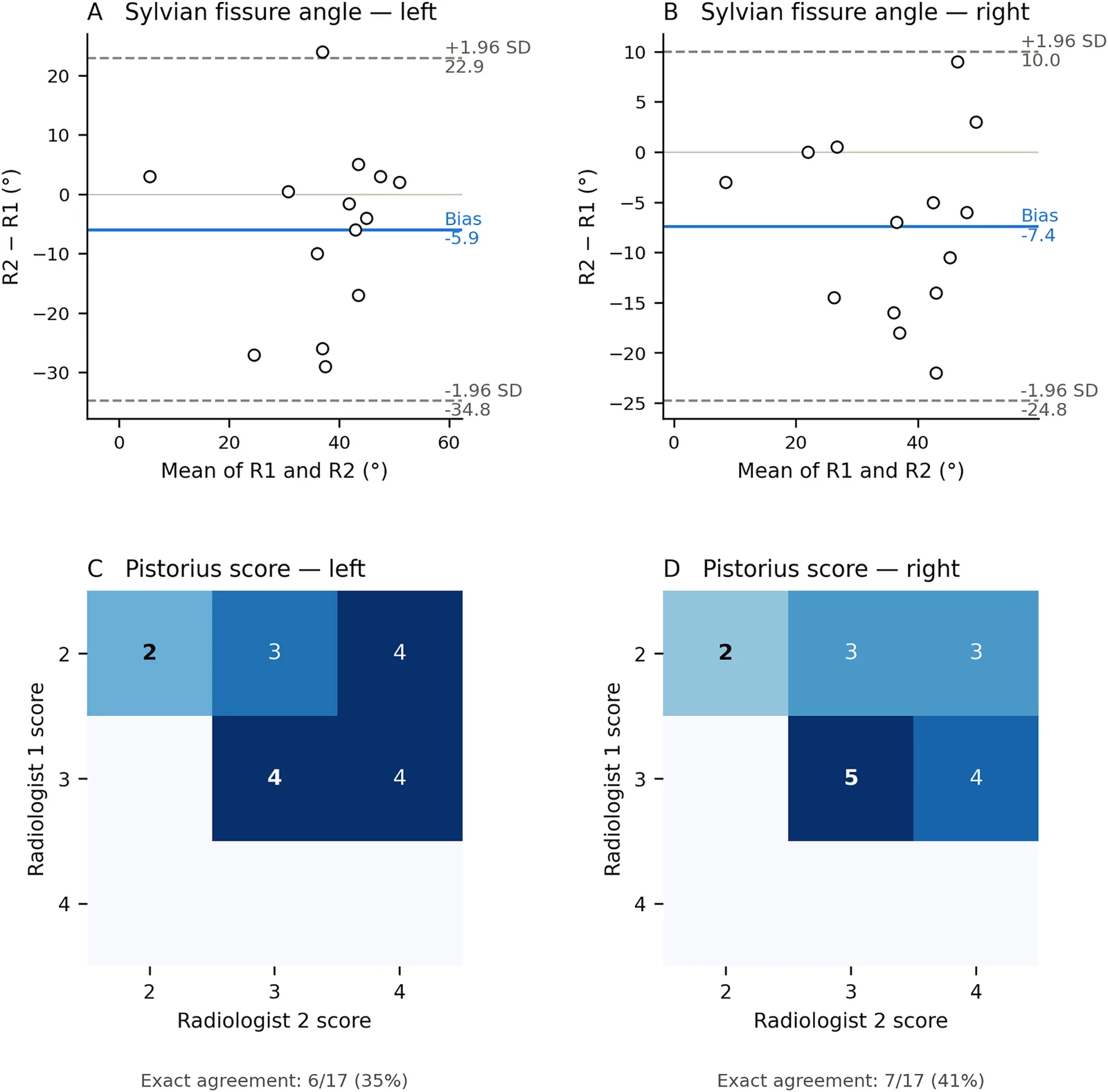
Inter-rater agreement between two radiologists for Sylvian fissure angle and Pistorius score measurements. Bland-Altman plots showing agreement between radiologist 1 (R1) and radiologist 2 (R2) for Sylvian fissure angle, left (A) and right (B); solid blue line indicates mean bias, dashed lines indicate 95% limits of agreement (bias ± 1.96 standard deviation). Agreement matrices showing paired R1/R2 Pistorius scores, left (C) and right (D); diagonal cells (bold) represent exact agreement between raters.

Fig. 2, Fig. 3, Fig. 4 show the Bland-Altman plots per radiologist using both methods (Fig. 2, Fig. 3) and between the radiologists for both methods (Fig. 4), with points colored by clinical subgroup (control, FGR without delayed maturation, FGR with delayed maturation) to allow visual inspection of subgroup-specific patterns. Overall, bias was low and the limits of agreement (LoA) relatively narrow. M2 with R1 yielded the best performance (bias +0.06 weeks; −0.75 to +0.88), though one R1/M1 case showed a marked deviation (−1.25 weeks) that widened the limits of agreement for M1 despite otherwise tight clustering, whereas R2 consistently overestimated GA and showed greater variability (up to +0.54 weeks). Despite the small mean biases, clinically relevant discrepancies between radiologists were frequent: R1 and R2 differed by ≥ 0.5 weeks in 12/18 cases for M1 and 14/20 for M2, and by ≥ 1 week in 7/18 and 8/20 cases, respectively. Differences between radiologists clustered at discrete 0.5-week intervals, reflecting the resolution at which GA estimates were recorded. No clear subgroup-specific pattern was apparent in the between-rater or rater-vs-actual comparisons, though the FGR subgroups were small (n = 5 each). Agreement for other markers was limited, with ICCs of 0.393 (left) and 0.649 (right) for the Sylvian fissure angle, and 0.098 (left) and 0.131 (right) for the Pistorius score. The distribution of Sylvian fissure angle and Pistorius score values by rater and subgroup is shown in Fig. 5; R2 had fewer valid measurements for both markers (Sylvian fissure angle, n = 14; Pistorius score, n = 17) due to motion artifact precluding visualization of the required plane in a subset of cases.

### 3.3 Delayed maturation

R1 identified delayed maturation in three cases using M1, including 2 healthy controls and 1 FGR case (−1.3, −0.8 and −0.6 weeks). R2 identified 2 delayed cases, both from the FGR cohort, with one overlapping case between R1 and R2 (−1.1 and −0.6 weeks). Using M2, R1 rated 2 FGR cases and 1 healthy control as delayed (−0.6, −0.6 and −0.5 weeks), while R2 identified 1 delayed FGR case (−0.6 weeks), which overlapped with R1. Only one fetal scan was consistently rated as delayed across all four assessments. Agreement metrics on delayed maturation are summarized in Table 1.

**Table 1:** Agreement on delayed maturation between radiologists and imaging methods.

	Concordance	Error	Cohen's Kappa	Interpretation
R1: M1 vs M2	80	20	0.22	Some agreement
R2: M1 vs M2	95	5	0.64	Substantial agreement
M1: R1 vs R2	85	15	0.32	Some agreement
M2: R1 vs R2	90	10	0.46	Moderate agreement

M1: Method 1, M2: Method 2, R1: Radiologist 1, R2: Radiologist 2.

When delayed maturation was reclassified as a wider 1-week threshold, the number of cases with delayed maturation decreased to 1/20 for R1/M1, 0/20 for R1/M2, 1/18 for R2/M1 and 0/20 for R2/M2. Concordance between methods and raters remained high (90-100%), but with so few cases the Cohen's kappa became unstable and for some comparisons paradoxically low or undefined (McHugh, 2012).

## 4 Discussion

By comparing two methods using a 2D template or 3D atlas for evaluating brain maturation on fetal MRI, we found that both yielded comparable results, supporting their potential clinical applicability. Our analysis focused specifically on brain maturation rather than brain biometry, addressing an area that remains underexplored but is highly relevant for antenatal neurodevelopmental assessment. Both radiologists achieved excellent ICCs across methods, with strong inter-rater agreement, indicating that the two approaches achieved comparably high reliability. These findings confirm our first hypothesis, that atlas-based assessment of fetal gyrification would demonstrate high reliability. In debriefing, radiologists reported that the first method was faster, whereas the second was more useful in cases where gestational age was difficult to determine. Nevertheless, despite overall strong agreement between the methods, diagnosing delayed maturation defined as and estimated GA≥0.5 weeks younger than the actual GA, remained challenging even with atlas-based approaches (Cohen's Kappa: 0.22-0.64). This finding is in line with our second hypothesis, that determining whether gyrification is delayed would prove difficult despite high overall reliability of the underlying GA estimation. The contrast between excellent ICCs for continuous GA estimation and only fair-to-moderate kappa values for the dichotomized delayed-maturation classification illustrates that reliability of a continuous measure does not necessarily extend to reliability of a categorical judgment derived from it. Small, non-systematic differences between raters and methods may have been sufficient to shift individual cases across the 0.5-week threshold. This likely reflects the small number of cases classified as delayed, the difficulty to distinguish between physiological variation and pathology, and the absence of a gold standard for validation. A further distinction must be made between the reliability and validity of this atlas-based approach. High agreement with actual GA, between methods and between raters confirm reliability within the cohort but does not establish that the method validly captures pathological delayed maturation. Notably, GA agreement remained high even within the FGR subgroup, and few FGR cases were classified as delayed, despite FGR being an established risk factor for impaired brain development (Meijerink et al., 2026). If FGR were consistently associated with delayed cortical maturation, GA estimates in these cases would be expected to systematically underestimate true GA; this was not clearly observed. This raises the possibility that visual, atlas-based gyrification assessment, may under-detect maturational delay in FGR, particularly given the small number of pathological cases evaluated. Overall, high reliability is a necessary but not sufficient step toward establishing clinical validity, and our findings should not be interpreted as evidence that the method accurately identifies true maturational delay.

### 4.1 Reliability in fetal brain maturation on MRI

To date, no studies have evaluated the reproducibility of gyrification or other specific maturation assessments on fetal MRI. In contrast, fetal brain biometry is a proxy for maturation with multiple studies reporting strong inter- and intra-rater agreement. Large cohorts of over 500 scans have shown ICCs exceeding 0.75–0.96 for most biometric parameters (Shi et al., 2020; Tilea et al., 2009; Verdera et al., 2023), although lower values have been reported for smaller structures such as the third and fourth ventricles and the corpus callosum (Frick et al., 2015). Recent studies using automated 3D reconstructions have also demonstrated high ICCs (>0.81) for 2D biometry, despite some variation between reconstruction software (Khawam et al., 2021; Brambilla et al.).

### 4.2 Other MRI techniques to assess cerebral maturation

This study focused on visual assessment of gyrification on T2-weighted imaging as a measure for brain maturation, given its clinical applicability. Myelination, another marker of maturation, may be indicated by T1 hyperintensity or reduced free motion of water on diffusion-weighted imaging (Garel et al., 2003). While diffusion MRI also provides insight into white matter development and microstructural integrity, no established gold standard exists for evaluating fetal myelination, which typically can be visualized on MRI from around 30 weeks of gestation (Garel et al., 2003; Christiaens et al., 2019; Parazzini et al., 2021).

An alternative approach for assessing cortical maturation is 3D cortical surface reconstruction, to delineate sulcal organization and calculate the fetal gyrification index (GI) (Clouchoux et al., 2012). Additional quantitative parameters, such as curvature, surface area, ridge detection, and tensor-based morphometry have been proposed to objectively characterize cortical folding on fetal MRI (Yehuda et al., 2023, 2024). Building on this, several studies have demonstrated that curvature- and shape-based descriptors of cortical morphology can accurately predict gestational age from fetal MRI, including in pathological cohorts (Ciceri et al., 2024b; Hong et al., 2021; Hu et al., 2013). However, these methods require 3D reconstruction and extensive post-processing, limiting their clinical utility. To address this, Yehuda et al. developed a semi-automated pipeline to extract cortical folding metrics from 2D T2-weighted images, defining five gyrification parameters: mean and maximum GI by area (GIA), mean and maximum GI by contour (GIC), and the bounded volume between the cortex and convex hull normalized to hemisphere volume. This approach effectively captured age-related gyrification changes and detected reduced folding in fetuses with lissencephaly and polymicrogyria (Yehuda et al., 2023). More recently, deep learning models applied to fetal MRI have achieved highly accurate fetal brain age predictions based on T2-weighted images with a mean absolute error of less than one day. This method is based on a single-center study and has not yet been clinically validated (Hong et al., 2021).

Taken together, these quantitative approaches offer objective, continuous measures of cortical folding and represent an important complementary direction to the visual, atlas-based approach evaluated here. However, their reliance on 3D surface reconstruction and specialized post-processing currently limits routine clinical implementation. The present study instead evaluated a pragmatic, observer-based method designed for direct clinical application without additional post-processing, and our findings should therefore be interpreted as evidence for the reliability of a clinically accessible screening approach, rather than as a substitute for the granularity offered by quantitative shape- or curvature-based analysis. Future work could usefully compare atlas-based visual assessment directly against these quantitative descriptors to determine whether the two approaches are concordant, and whether quantitative methods could help resolve the classification difficulty observed for delayed maturation in the present study.

### 4.3 Strengths & limitations

A strength of the study was that it was blinded and assessed independently by two pediatric radiologists with varying years of experience, which reduces potential bias and enhances the reliability of our findings. Secondly, reliability was quantified in ICC values, providing objective evidence of reproducibility. Thirdly, as this topic has not been systematically studied before, our findings help to optimize clinical methods and allow a more standardized interpretation of gyrification on fetal MRI in daily practice.

However, several limitations should be acknowledged. The absence of a gold standard for fetal brain maturation on MRI limits the validation of our findings. This limitation is particularly relevant given that the high reliability observed in this study, including within the FGR subgroup, should not be interpreted as evidence of the method's validity for detecting pathological delayed maturation since we recently illustrated that FGR is associated with delayed gyrification (Meijerink et al., 2026). Furthermore, the cohort was not matched by gestational age at scan: FGR cases were scanned significantly earlier than healthy controls (median 29.1 vs. 31.7 weeks), reflecting that FGR scans were performed clinically following diagnosis, whereas control scans were performed at a fixed research timepoint. This GA imbalance means the present study should not be interpreted as a matched case-control comparison and limits our ability to disentangle true group differences in maturation from differences attributable to GA at scan itself; findings within the FGR subgroup should therefore be interpreted with caution. Additionally, the methods are based on the fetal brain MRI atlas by Gholipour et al. (2017), which represents an average anatomical template derived from 81 healthy fetuses between 19 and 39 weeks of gestation. While valuable for standardization, this atlas consequently limits the ability to capture individual variability in gyrification, which is known to occur (Gholipour et al., 2017; Volpe et al., 2017). Although the radiologists were blinded for all clinical information except gestational age to mimic clinical practice, not blinding gestational age could be considered a limitation. Knowledge of the actual GA likely anchored radiologists' estimates toward the known value, inflating the observed agreement and potentially reducing sensitivity to true maturational delay. This offers a complementary explanation for the FGR paradox discussed above: high GA agreement in FGR cases may partly reflect anchoring rather than true absence of delay. GA-informed assessment was retained to mirror clinical practice but limits our ability to distinguish true reliability from agreement driven by anchoring. Similarly, the fixed, non-randomized order in which method 1 and method 2 were performed introduces a potential order bias; although assessments were separated by at least one week to reduce recall of prior ratings, residual familiarity with a case's imaging characteristics may still have influenced the second evaluation. Despite the high ICC values, assessments were still based on subjective interpretation, rather than objective measurements. Most importantly, the small sample size and very limited number of cases with suspected delayed maturation substantially restrict the statistical power and limit generalizability, even though this sample is adequate for a reliability study.

## 5 Conclusion & recommendation

This study demonstrates that gestational age can be estimated with high reliability, both in agreement with actual GA and between raters, by assessing gyrification using both 2D and 3D MRI templates from the Gholipour fetal brain atlas(Gholipour et al., 2017), with consistent performance across pediatric radiologists with varying levels of experience. The 2D method (method 1) was less time-consuming and more consistent, whereas the 3D method (method 2) was considered more useful in cases where gestational age was difficult to determine. These findings support the reliability of standardized fetal brain templates within this single-center cohort; however, this does not establish reproducibility across centers, scanners, protocols, or clinical settings, nor does reliability alone establish the clinical validity of this approach for detecting pathological delayed maturation.

Moreover, the assessment of delayed gyrification remains challenging, as there is currently no gold standard to confirm this finding. Despite the small mean biases between raters, clinically relevant discrepancies were common: R1 and R2 estimates differed by ≥ 0.5 weeks in 12/18 cases for M1 and 14/20 for M2, and by ≥ 1 week in 7/18 and 8/20 cases, respectively. These discrepancies are directly relevant to the proposed 0.5-week classification threshold and should be weighed when considering the potential clinical applicability of these methods. While this study provides initial insights into the reliability of atlas-based gyrification assessment, further investigation is needed with larger and more diverse cohorts, ideally confirming cases of delayed cortical maturation with postnatal MRI or histopathologic validation.

Future research should link fetal gyrification to neurodevelopmental outcomes and prioritize the development and clinical translation of automated deep learning models to improve reproducibility and objectivity.

## Generative AI declaration

During the preparation of this manuscript, the authors used Claude (Anthropic) and ChatGPT (OpenAI) to improve the readability and phrasing of selected sentences. These tools were not used in the study design, data collection, analysis, interpretation of results, or generation of scientific content. After using these tools, the authors reviewed and edited the content as needed and take full responsibility for the content of the publication.

## CRediT authorship contribution statement

**Lotte Meijerink:** Conceptualization, Data curation, Formal analysis, Investigation, Methodology, Project administration, Software, Visualization, Writing – original draft. **Yvette Stam:** Conceptualization, Data curation, Methodology, Writing – review & editing. **Fieke Terstappen:** Conceptualization, Methodology, Supervision, Writing – review & editing. **Thomas Alderliesten:** Conceptualization, Methodology, Supervision, Writing – review & editing. **Manon Benders:** Conceptualization, Supervision, Writing – review & editing. **Wouter P. Nieuwenhuis:** Conceptualization, Formal analysis, Resources, Validation, Visualization, Writing – review & editing. **Rutger A.J. Nievelstein:** Conceptualization, Formal analysis, Resources, Validation, Visualization, Writing – review & editing. **Mireille Bekker:** Conceptualization, Supervision, Writing – review & editing.

## Declaration of competing interests

The authors declare that they have no known competing financial interests or personal relationships that could have appeared to influence the work reported in this paper.

## Data Availability

The data that has been used is confidential.
